# Bioinformatic analysis reveals *MIR502* as a potential tumour suppressor in ovarian cancer

**DOI:** 10.1186/s13048-020-00683-y

**Published:** 2020-07-13

**Authors:** Yan Li, Qi Wang, Ning Ning, Fanglan Tang, Yan Wang

**Affiliations:** Department of Obstetrics and Gynecology, The First Affiliated Hospital of Harbin Medical University, 23 Youzheng Street, Nangang District, Harbin, Heilongjiang China

**Keywords:** *MIR502*, NRF1, Hippo signalling pathway, Ovarian cancer

## Abstract

**Background:**

Ovarian cancer (OC) is a major cause of death among women due to the lack of early screening methods and its complex pathological progression. Increasing evidence has indicated that microRNAs regulate gene expression in tumours by interacting with mRNAs. Although the research regarding OC and microRNAs is extensive, the vital role of *MIR502* in OC remains unclear.

**Methods:**

We integrated two microRNA expression arrays from GEO to identify differentially expressed genes. The Kaplan–Meier method was used to screen for miRNAs that had an influence on survival outcome. Upstream regulators of *MIR502* were predicted by JASPAR and verified by ChIP-seq data. The LinkedOmics database was used to study genes that were correlated with *MIR502*. Gene Set Enrichment Analysis (GSEA) was conducted for functional annotation with GO and KEGG pathway enrichment analyses by using the open access WebGestalt tool. We constructed a PPI network by using STRING to further explore the core proteins.

**Results:**

We found that the expression level of *MIR502* was significantly downregulated in OC, which was related to poor overall survival. NRF1, as an upstream regulator of *MIR502*, was predicted by JASPAR and verified by ChIP-seq data. In addition, anti-apoptosis and pro-proliferation genes in the Hippo signalling pathway, including CCND1, MYC, FGF1 and GLI2, were negatively regulated by *MIR502*, as shown in the GO and KEGG pathway enrichment results. The PPI network further demonstrated that CCND1 and MYCN were at core positions in the development of ovarian cancer.

**Conclusions:**

*MIR502*, which is regulated by NRF1, acts as a tumour suppressor gene to accelerate apoptosis and suppress proliferation by targeting the Hippo signalling pathway in ovarian cancer.

## Introduction

Ovarian cancer (OC) is a common malignancy with the highest mortality rate among all gynaecological tumours [1]. Primary cytoreductive surgery following chemotherapy is the conventional treatment of OC. These tumours often exhibit extensive proliferation, invasion, and lymph node metastasis at the time of diagnosis due to the lack of typical symptoms in the early stage, which leads to a delay in initiating appropriate treatment and poor outcomes [2]. The pathogenesis of OC is complicated because it is regulated by a variety of oncogenes and tumour suppressor genes [3]. Currently, multiple ovarian cancer oncogenes have been elucidated, whereas relatively few studies have focused on antioncogenes, and the molecular mechanisms regulating the progression of OC remain mostly unclear. Therefore, it is of considerable importance to explore new molecular pathways regulating the proliferation and apoptosis of ovarian cancer cells and to provide potential targets for clinical treatment.

microRNAs (miRNAs) are small RNA molecules with a length of approximately 20 nucleotides, whose function is negatively regulating gene expression at the post-transcriptional level through binding to the 3′-untranslated regions (3′-UTRs) of target mRNAs [4, 5]. A substantial amount of research has confirmed that multiple miRNAs played pivotal roles in the process of tumour development, regulating apoptosis, proliferation, invasion, migration and recurrence by reducing or increasing the expression of various proteins [6, 7]. In particular, various miRNAs have been shown to have different roles in ovarian cancer. However, research into the regulatory mechanisms and target genes of miRNAs is still in its infancy, and the relationship between miRNAs and tumours, especially ovarian cancer, is not fully understood. Currently, the effect of *MIR*502 in cancer has been researched widely. The results from our study indicated that *MIR*502 had a marked effect on suppressing ovarian cancer proliferation.

Nuclear respiratory factor 1 (NRF-1) is an important transcription factor in the human genome. A systematic bioinformatics study estimated that 6% of human promoter region genes contain NRF-1 response elements [8]. NRF-1, also known as a-pal, was initially identified as a mitochondrial gene involved in the regulation of energy conduction [9]. NRF1 encodes a protein that forms a homologous dimer and acts as a transcription factor, regulating the expression of some key metabolic genes regulating cell growth [10].

The Hippo signalling pathway has a crucial role in regulating cell proliferation, regeneration and controlling organ growth [11]. This pathway is comprised of a large number of proteins. It has the function of controlling cell fate not only in the process of development and differentiation but also in pathological processes, including cancer [12]. The main Hippo transcriptional coactivators are Yes-associated protein (YAP) and transcriptional coactivator with the PDZ-binding motif (TAZ). There is a strong relationship between Yap activation and cancer. In many tumours, including those of the brain, lung, breast, pancreas, liver, colon, skin and ovary, YAP and TAZ promote cell proliferation and anti-apoptosis in cooperation with transcription factors by translocating into the nucleus to regulate many well-known oncogenes [13–17]. A study on podocytes found that YAP overexpression led to CCND1 being significantly upregulated, which confirmed CCND1 as a downstream target gene of YAP [18]. Previous research on gastric tumours has identified MYC as a key downstream molecular target of YAP. The positive correlation between MYC and YAP in human gastric cancers also supports the regulation of MYC by YAP, which is an important molecular mediator of gastric tumourigenesis [19]. Shan Xu has verified that YAP promoted VEGFA by targeting GLI2 in renal cancer [20]. Some studies have shown that FGF promotes Hippo/Yap signal transduction in the proliferation and differentiation processes of lens epithelial cells, and FGF-induced nuclear Yap expression plays an important role in promoting lens epithelial cell proliferation [21]. Accordingly, it has been reported that YAP, acting as an oncogene, is associated with a poor prognosis of ovarian cancer [22–24].

The big data generated by high-throughput research is generally characterized by its large amount, a wide range of data types, deep value mining and fast processing responses. Big data provides opportunities for the discovery of tumour molecular targets, but it also brings great challenges to the full mining, integration and utilization of the results. Investigation of complex genetic mechanisms by applying the appropriate statistical method is certainly needed [25].

In this study, we found that the expression level of *MIR*502 was significantly down-regulated in ovarian cancer by using bioinformatics analysis of two public databases. We also analysed the association of *MIR*502 expression with overall survival (OS), and correlated pathways were explored to provide prognostic and therapeutic value in preventing ovarian cancer progression. Gene Ontology (GO) and Kyoto Encyclopedia of Gene and Genomes (KEGG) pathway analysis showed that the Hippo signalling pathway was correlated with *MIR*502. The transcription factor NRF1 was predicted as an upstream regulator of *MIR502*. The authors believe that these findings may provide more effective and scientific guidance to clinicians for the early diagnosis of patients with ovarian cancer, along with individualized treatment, and improve the prognosis of the patients.

## Materials & methods

### Accession of the public database

The microRNA expression datasets used in this study (GEO: GSE83693 and GSE119055) were acquired from the National Centre for Biotechnology Information (NCBI) Gene Expression (http://www.ncbi.nlm.nih.gov/geo/).

### Analysis of the public database

GEO2R (http://www.ncbi.nlm.nih.gov/geo/geo2r/) is an analysis tool that is used to compare two sets of data coming from the GEO database. We used GEO2R to screen for differentially expressed miRNAs between healthy ovarian tissue and ovarian cancer tissue in the GSE83693 and GSE119055 datasets. We selected genes whose |log2FC (fold change)| > 2 and adjusted *P*-value < 0.05 as differentially expressed genes.

### Survival analysis

According to the lower quartile expression level of *MIR502*, the OC patients were divided into a high expression group and a low expression group. The overall survival was analysed by using Kaplan–Meier plotter (http://kmplot.com/analysis/index.php?p=background). The hazard ratio with 95% confidence intervals and log-rank *P*-value were calculated and displayed.

### Gene correlation expression analysis

The LinkedOmics database (http://www.linkedomics.org/admin.php) contains 32 TCGA cancer-associated multi-dimensional datasets, including ovarian cancer. This website was used to study the correlation between *MIR*502 and the expression of the genes of interest in the TCGA OC cohort. The results were analysed statistically using Pearson’s correlation coefficient.

### Prediction and verification of transcription factors

We used JASPAR (http://jaspar.genereg.net) to predict the transcription factors of CLCN5, and the Cistrome Data Browser (http://cistrome.org/db/) as a resource of human cis-regulatory information obtained from chromatin analysis from ChIP-seq, DNase-seq and ATAC-seq. It was used to verify the prediction results.

### Acquisition of overexpressed genes of ovarian cancer from the cancer genome atlas database

The Gene Expression Profiling Interactive Analysis (GEPIA) website (http://gepia.cancer-pku.cn) can provide varied functions based on TCGA data, including gene expression, gene correlation analysis, survival analysis, and so on. GEPIA was used to find overexpressed genes in ovarian cancer. *P* < 0.05 was considered statistically significant.

### GO and KEGG pathway analysis

Gene Set Enrichment Analysis (GSEA) was conducted for functional annotation with GO and KEGG pathway enrichment analyses by using the open access WebGestalt tool (http://www.webgestalt.org). GO analysis included biological process (BP), cellular component (CC) and molecular functions (MF). The results with a false discovery rate (FDR) ≤0. 05 were considered noteworthy.

### Target genes prediction of *MIR502*

miRWalk (http://zmf.umm.uni-heidelberg.de/apps/zmf/mirwalk/micrornapredictedtarget.html) was applied to forecast the genes targeted by *MIR*502. In total, five servers with DIANA-mT, miRanda, miRWalk, PICTAR5 and Targetscan were used. Only those genes projected by all five servers were selected as target genes.

### Protein-protein interaction network construction

The protein-protein interaction (PPI) network was constructed based on the overlapping genes that appeared in the predicted genes in miRWalk and in the overexpressed genes in GEPIA by using the Search Tool for the Retrieval of Interacting Genes (STRING, version 11.0, https://string-db.org/) database.

### Statistical analysis

Statistical analysis was performed using Prism software (GraphPad, CA, USA). The statistical significance of differences between and among groups was assessed using the t-test. Significant differences are indicated as follows: ∗*P* < 0.05; ∗∗*P* < 0.01; ∗∗∗*P* < 0.001.

## Results

### The expression level of *MIR502* was lower in ovarian cancer tissue compared with healthy ovary tissue

**Fig. 1:**
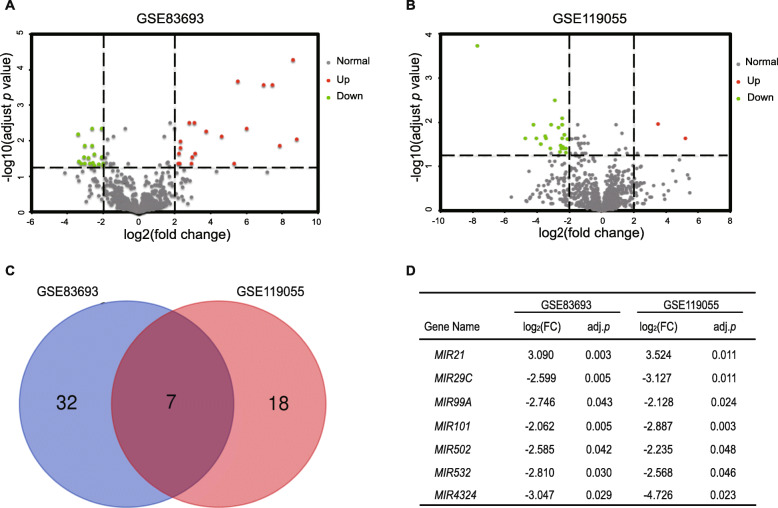
The expression level of *MIR502* is lower in ovarian cancer tissue comparing with normal ovary tissue. **a-b** Volcano plots of detectable genome-wide miRNA profiles in ovarian cancer tissue and normal ovarian tissue samples from GSE83693 and GSE119055, respectively. Green and red plots represent aberrantly expressed miRNAs with *P* < 0.05 and |log2(FC)| > 2. Green plots indicate downregulated genes, red plots indicate upregulated genes, and grey plots indicate normally expressed miRNAs. **c** Venn diagram of GSE83693 and GSE119055, **d** Detailed information of seven common different expression miRNAs are listed

To explore the difference in microRNAs expression in human ovarian cancer tissue, we obtained two microarray gene profiling datasets (GSE83693 and GSE119055) from the public GEO datasets of NCBI. Detailed information about the two datasets is shown in Table 1. After analysing the expression of the microRNAs, we screened out 39 and 25 differentially expressed genes (DEGs) from the GSE83693 and GSE119055 datasets, respectively, which are shown in volcano plots (Fig. 1a, b). Seven common DEGs were screened out with Bioinformatics and Evolutionary Genomics (http://bioinformatics.psb.ugent.be/webtools/Venn/) (Fig. 1c) and listed in Fig. 1d.

**Table 1:** Features of the enrolled datasets

Accession	GPL	Year	Samples		Source
			Control	OC	
GSE83693	GPL22079	2017	4	8	tissue
GSE119055	GPL21572	2019	3	6	tissue

*OC* Ovarian cancer

### Expression of *MIR502* was correlated with the overall survival of the OC patients

**Fig. 2:**
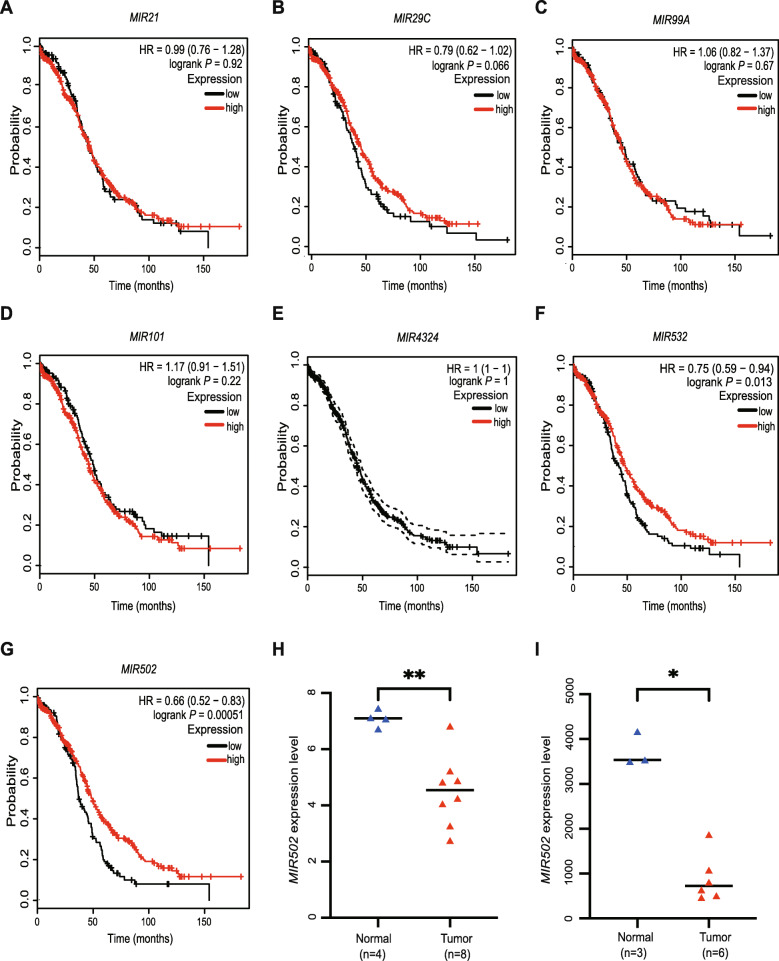
Expression of *MIR502* affected the overall survival of OC patients. Kaplan-Meier analysis of overall survival (OS) in OC patients based on the K-M plotter dataset. **a-e***MIR21*, *MIR29c*, *MIR99a*, *MIR101* and *MIR4324* expression is not correlated with OS in OC patients. **f***MIR532* is positively correlated with OS in OC patients, *P* < 0.05. **g***MIR502* is positively correlated with OS in OC patients, *P* < 0.01. **h** The expression level of *MIR502* in normal and ovarian cancer tissues from GSE83693 (normal tissues, *n* = 4; OC tissues, *n* = 8, *P* < 0.01). I The expression level of *MIR502* in normal and ovarian cancer tissues from GSE119055 (normal tissues, *n* = 3; OC tissues, *n* = 6, *P* < 0.05). **P < 0.01, *P < 0.05

After analysing the overall survival of the OC patients by the Kaplan–Meier method, we found that only the expression levels of *MIR*502 (*p* < 0.01) and *MIR*532 (*p* = 0.013) among seven microRNAs were correlated with the overall survival outcome (Fig. 2a–g). Research into the role of *MIR*532 in ovarian cancer has made headway, but relatively few studies have explored the mechanism of *MIR*502 in OC, so our main focus of study was *MIR*502. We present a box-plot to show the expression of *MIR*502 in each database (Fig. 2h, i).

### Genes correlated with *MIR502* in ovarian cancer

**Fig. 3:**
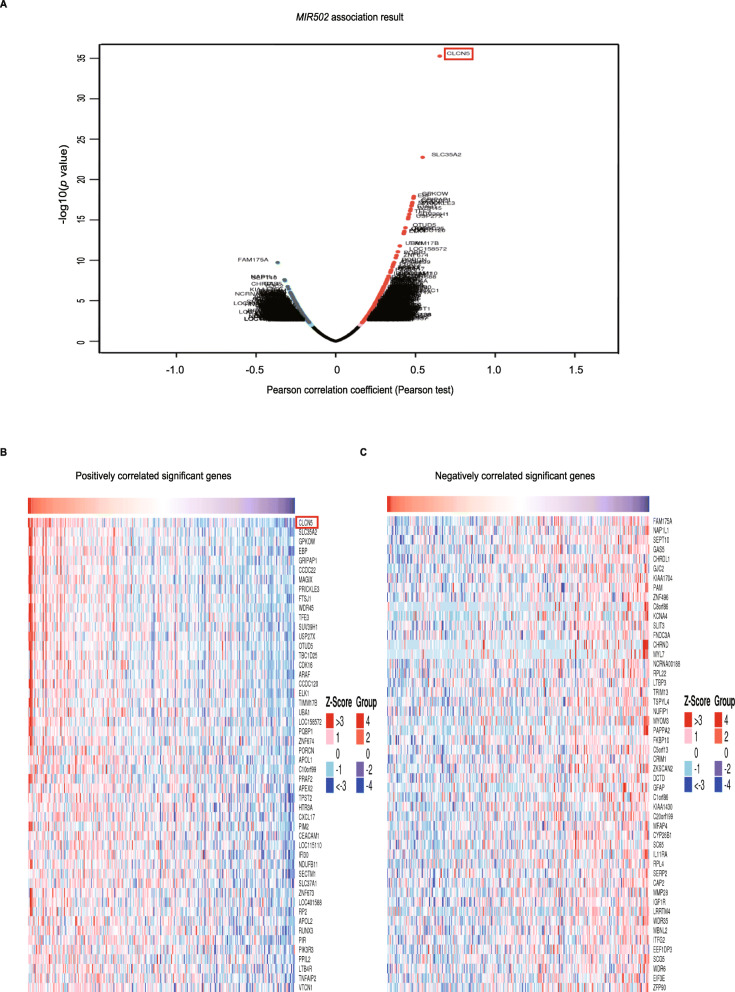
Genes correlated with *MIR502* in ovarian cancer. **a** Pearson test was used to analyse correlations between *MIR502* and genes differentially expressed in ovarian cancer. **b-c** Heat maps showing genes positively and negatively correlated with *MIR502* in ovarian cancer (TOP 50). Red indicates positively correlated genes and blue indicates negatively correlated genes

The volcano plot shows genes positively and negatively correlated with *MIR*502 (Fig. 3a). The top 50 significant gene sets with positive and negative correlations with *MIR*502 are shown in the heat map (Fig. 3b, c). The heat map demonstrates a widespread influence of *MIR*502 on the transcriptome.

### *MIR502* is closely related to CLCN5

**Fig. 4:**
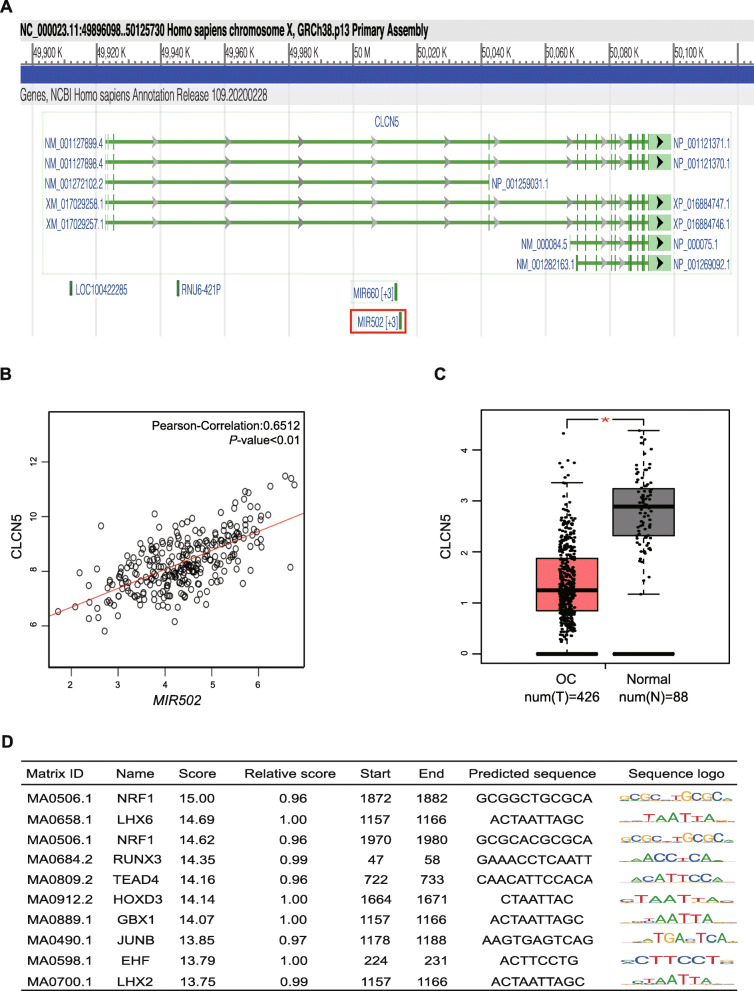
*MIR502* was closely related to CLCN5. **a***MIR502* hosted in the CLCN5 gene. **b** Correlation of the expression levels of *MIR502* and CLCN5. **c** Expression level of CLCN5 in ovarian cancer (number = 426) and normal ovary tissues (number = 88), *P* < 0.05. D Predicted transcription factors of CLCN5

CLCN5 showed the strongest positive correlation with *MIR*502, as shown in Fig. 3b and Fig. 4b (Pearson-correlation = 0.6512, *P* < 0.01). For further exploration, by searching NCBI we found *MIR*502 was hosted in the third intron of the CLCN5 gene (Fig. 4a). The expression level of CLCN5 in OC was lower than that in healthy ovarian tissue (Fig. 4c), which is consistent with the expression pattern of *MIR*502. The JASPAR (http://jaspar.genereg.net/) database was used to analyse and predict the transcription factors that potentially regulated the expression of CLCN5. By matching the 2000 bp region of the nucleotide sequence upstream of the promoter of the CLCN5 gene, we found transcription factor NRF1 was the highest matched (Fig. 4d).

### NRF1 acts as a transcription factor of CLCN5

**Fig. 5:**
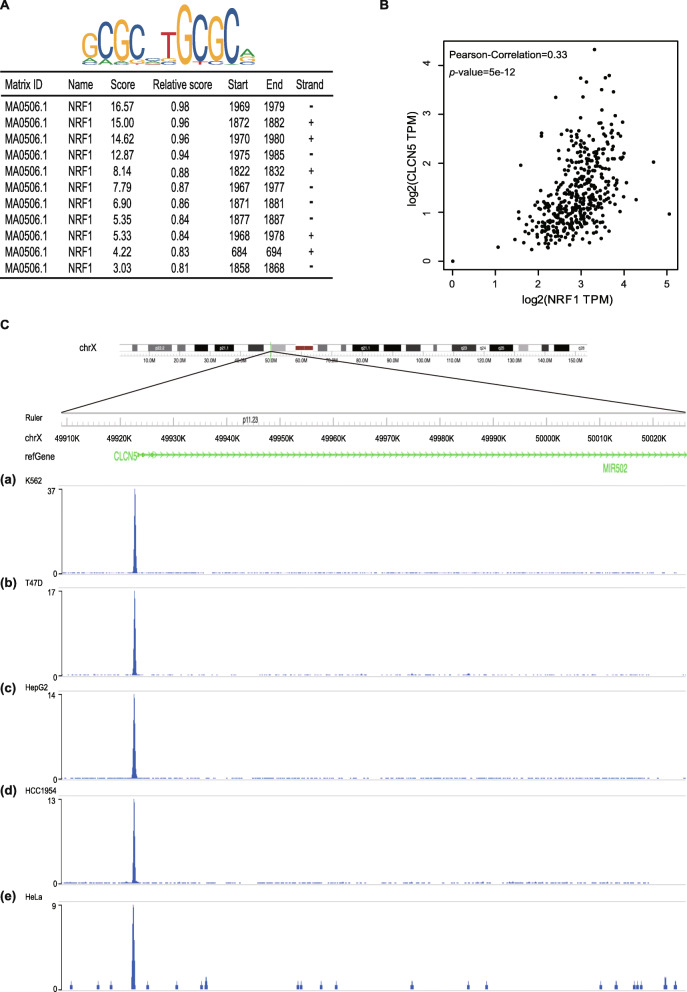
NRF1 acted as a transcription factor of CLCN5. **a** The upper part of the picture shows the NRF1 binding sequence, and the lower table shows the prediction of NRF1 binding sites within the promoter region of CLCN5 provided by the JASPAR database. **b** Positive correlation of the expression levels of CLCN5 and NRF1. **c** Analysis of CLCN5 ChIP-seq data from K562, T47D, HepG2, HCC1954 and HeLa cells at the CLCN5 promoter from Cistrome Data Browser databases

The match score and binding site of NRF1 are shown in Fig. 5a. The expression of NRF1 is positively correlated with CLCN5 (Pearson-correlation = 0.33, *P* < 0.01) (Fig. 5b). We used the Cistrome Data Browser (http://cistrome.org/db) database to analyse the ChIP-seq data of tumour cells, and we found that NRF1 had a DNA binding peak in the promoter region of CLCN5 (Fig. 5c). This further confirmed that NRF1 binds to and regulates CLCN5 expression as a transcription factor.

### GO and KEGG pathway analysis of genes correlated with *MIR502* in ovarian cancer

**Fig. 6:**
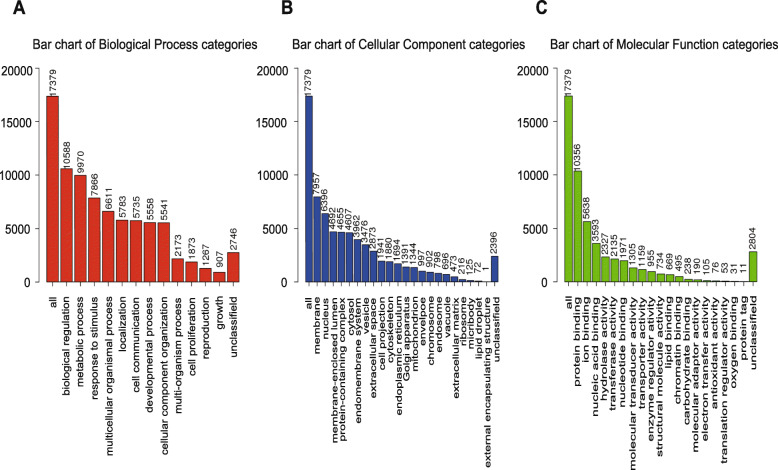
GO term analysis of correlated genes with MIR502 in ovarian cancer. **a** For biological process categories. **b** For cellular component categories. **c** For molecular function categories

**Fig. 7:**
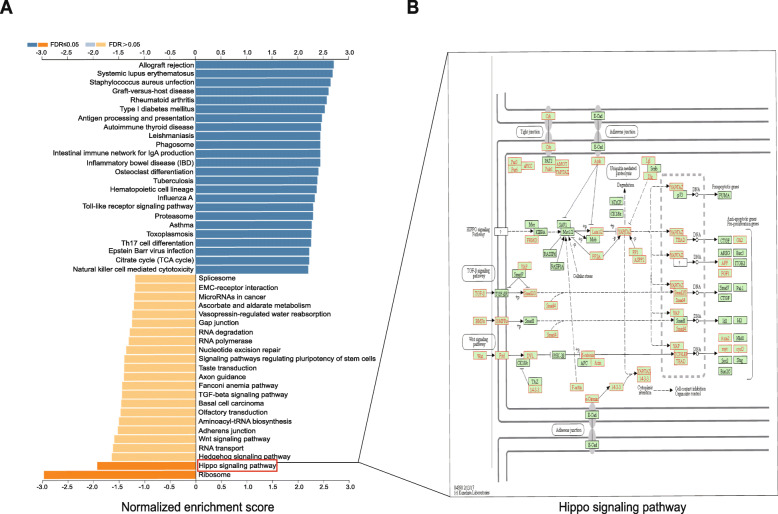
KEGG pathway analysis and Hippo signalling pathway. **a** Bar of KEGG analysis of *MIR502* correlated genes-associated pathways. **b** Hippo signalling pathway diagram

**Fig. 8:**
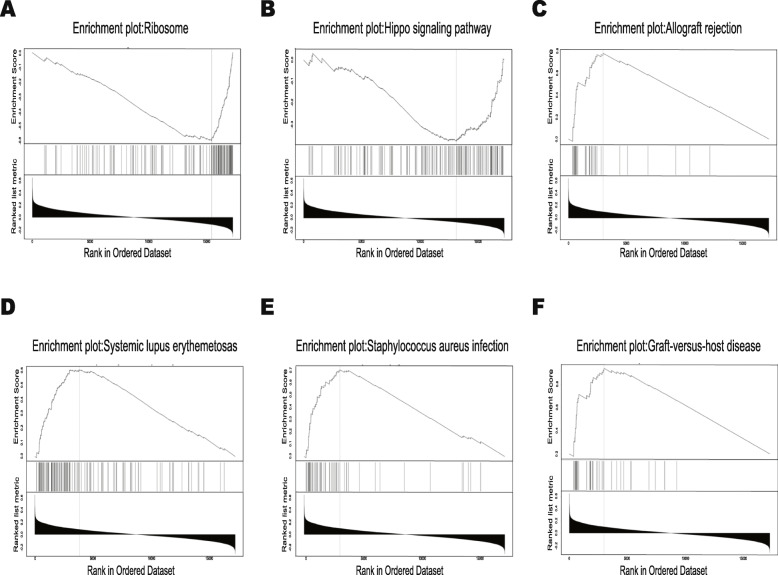
GSEA analysis of *MIR502* correlated expressed genes. **a** Ribosome, NSE = -2.728, *P* = 0. **b** Hippo signalling pathway, NSE = -1.7788, *P* = 0. **c** Allograft rejection, NSE = 2.188, *P* = 0. **d** Systemic lupus erythematosus, NSE = 2.255, *P* = 0. **e** Staphylococcus aureus infection, NSE = 2.208, *P* = 0. **f** Graft-versus-host disease, NSE = 2.149, *P* = 0

GO term analysis was given a broad overview by using Go Slim (Fig. 6). The results indicated that these genes could be categorized into several important biological processes, including biological regulation, metabolic process, membrane, nucleus, protein binding and ion binding. Significant GO terms were examined in more detail by GSEA, showing that genes correlated with *MIR*502 were located mainly in protein localization to the endoplasmic reticulum (GO:0070972) and translational initiation (GO:0006413) for BP, ribosome (GO:0005840) and tertiary granule (GO:0070820) for CC, and structural constituent of ribosome (GO:0003735) and pattern recognition receptor activity (GO:0038187) for MF (Table 2). The KEGG pathway analysis showed that the correlated genes were enriched in various pathways (Fig. 7a), including ribosome, allograft rejection pathways, systemic lupus erythematosus, and so on. It should be noted that the Hippo signalling pathway also appeared in the enrichment results. A detailed signalling pathway diagram is shown in Fig. 7b. The genes correlated with *MIR*502 are marked in red. The significant enrichment results are shown in Fig. 8.

**Table 2:** Enriched GO and KEGG items

Enriched Category	Description	Count	NES	*P*-Value	FDR
**Biological process**					
GO:0070972	protein localization to endoplasmic reticulum	135	−2.628	0	0
GO:0006413	translational initiation	179	−2.581	0	0
GO:0034341	response to interferon-gamma	187	2.395	0	0
**Cellular components**					
GO:0005840	ribosome	216	−2.309	0	0
GO:0070820	tertiary granule	155	2.238	0	0
GO:0042581	specific granule	152	2.129	0	0
**Molecular function**					
GO:0003735	structural constituent of ribosome	152	−2.666	0	0
GO:0038187	pattern recognition receptor activity	20	1.992	0	0.009
GO:0019843	rRNA binding	58	−1.971	0	0.004
**KEGG pathway**					
hsa03010	Ribosome	129	−2.728	0	0
hsa05322	Systemic lupus erythematosus	122	2.255	0	0
hsa04390	Hippo signaling pathway	148	−1.778	0	0.035

Table shows three items each from GO-BP, GO-CC, GO-MF and KEGG

### *MIR502* regulated CCND1, FGF1, MYC and GLI2

**Fig. 9:**
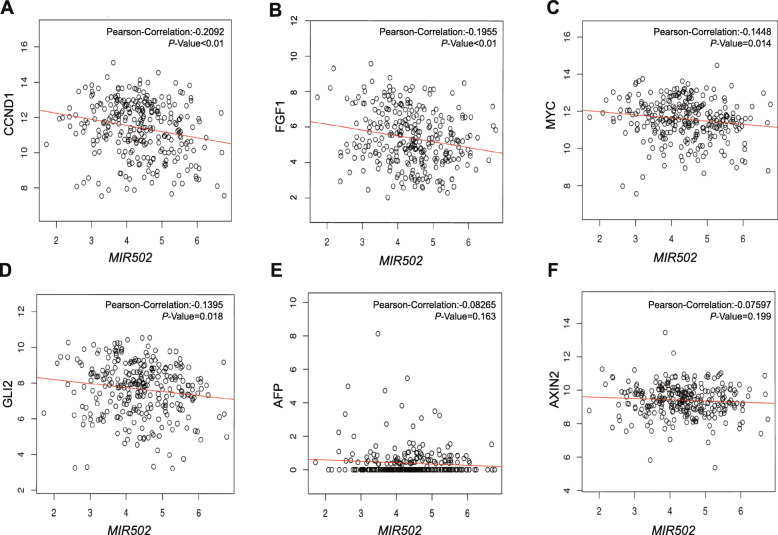
*MIR502* regulated CCND1, FGF1, MYC and GLI2. Correlation of the expression levels of *MIR502* and Hippo signalling pathway downstream genes, including CCND1, FGF1, MYC, GLI2, AFP and AXIN2. Data were analysed using Pearson’s R correlation. *, *P* < 0.05; **, *P* < 0.01; ***, *P* < 0.001

Our study showed that six well-characterized genes with the functions of anti-apoptosis and pro-proliferation participated in the Hippo signalling pathway, including CCND1, FGF1, MYC, GLI2, AFP and AXIN2 (Fig. 7b). The LinkedOmics database was used to confirm the correlation between the six genes and *MIR*502. The results indicated that *MIR*502 negatively regulated CCND1 (Pearson-correlation = − 0.2092, *p* < 0.01), FGF1 (Pearson-correlation = − 0.1955, *p* < 0.01), MYC (Pearson-correlation = − 0.1448, *p* < 0.05) and GLI2 (Pearson-correlation = − 0.1395, *p* < 0.05) (Fig. 9).

### CCND1 and MYCN were at core positions in the PPI network

**Fig. 10:**
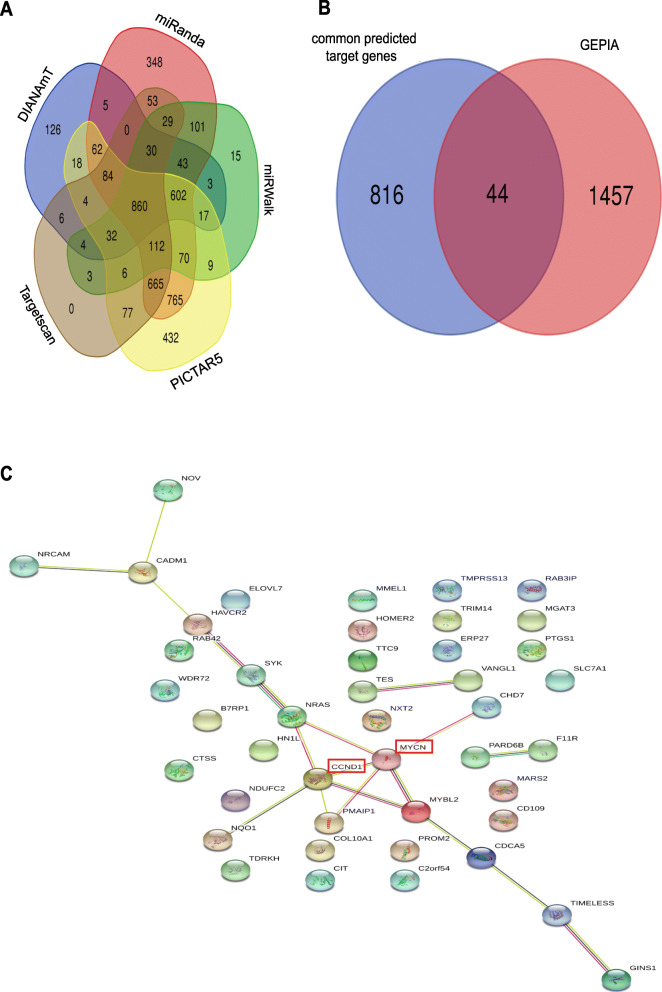
CCND1 and MYCN were at the core position in the PPI network. **a** Venn diagram of predicted target genes of *MIR502* by using miRanda, miRWalk, PICTAR5, Targetscan and DIANAmT, 860 common genes were selected. **b** Venn diagram of 860 common predicted target genes and 1501 overexpression genes in ovarian cancer obtained from GEPIA, 44 common genes were selected as hub genes. **c** The protein–protein interaction networks of 44 hub genes of *MIR502* in ovarian cancer. Nodes represent gene-encoded proteins. Connections between nodes represent the relationship between proteins. A bolder line implies a higher confidence level

The 860 common genes were selected as predicted target genes of *MIR502* (Fig. 10a). A total of 44 genes were selected in the overlapping areas of the 860 predicted target genes and 1501 overexpressed genes in the GEPIA of ovarian cancer (Fig. 10b). The PPI network revealed CCND1 and MYCN at the core position (Fig. 10c). NRAS, PMAIP1 and MYBL2 showed interaction relationships with both CCND1 and MYCN.

## Discussion

The aim of our study was to identify miRNAs that were obviously differentially expressed in OC tissue compared with normal tissue and to improve ovarian cancer patients’ overall survival by exploring the mechanisms regulating particular pathways. We selected *MIR*502 as our main miRNA after screening miRNAs through a strict selection process. Our survival analysis showed that *MIR*502 conferred a protective phenotype to OC patients, with a higher expression of *MIR*502 predicting a longer overall survival. *MIR*502 is located in the third intron of the CLCN5 gene, and it shows a strong positive correlation with CLCN5 in ovarian cancer. We predicted NRF1 as a transcription factor regulating CLCN5, and ChIP-seq data of various tumour cells verified the binding peak between NRF1 and CLCN5. We demonstrated that NRF1, as a transcription factor regulating CLCN5, regulated the expression of *MIR502* indirectly, which clarified the upstream regulatory mechanism of *MIR*502.

To explore the downstream regulatory mechanism of *MIR*502 in ovarian cancer, we further predicted and analysed genes correlated with *MIR*502. We identified a set of biological functions and related signalling pathways that *MIR*502 might regulate in ovarian cancer. Furthermore, the GSEA annotation analysis results showed that *MIR*502 negatively regulated anti-apoptosis and pro-proliferation genes, such as CCND1, FGF1, MYC, and GLI2, in the Hippo signalling pathway. All of these results demonstrated that the expression of *MIR502* was down-regulated in OC, which increased the expression levels of the oncogenes CCND1, FGF1, MYC and GLI2, which have important functions in anti-apoptosis and promote the development of OC. The PPI network also suggested that CCND1 and MYCN were both target genes regulated by *MIR502*, and they were at the centre position of interaction with other proteins.

CCND1, also known as cyclin D1, is a member of the cell cycle family of proteins [26]. CCND1 regulates cell cycle progression by promoting the cell cycle transition from G1 to S phase [27–29]. The abnormal expression of CCND1 promotes cell proliferation by regulating the cell cycle [30]. Previous researchers have demonstrated that CCND1, identified as a proto-oncogene, has an essential role in the development of many kinds of tumours, including lung adenocarcinoma, glioma and renal cell cancer [31–33]. In addition, some studies have shown that overexpression of CCND1 promotes tumour cell invasion and metastasis in breast cancer and gastric cancer, leading to a poor prognosis [34, 35]. Compared with that in normal tissues, the expression of CCND1 is obviously higher in bladder cancer tissues, reproductive system tumours, gastric cancer tissues and lung cancer tissues, and it is correlated with the pathological type and clinical stage of the tumour [36–38]. CCND1 expression is closely related to cell proliferation ability and apoptosis in epithelial ovarian cancer cells. A study of epithelial ovarian cancer observed that overexpression of CCND1 leads to stronger cell growth ability and less apoptosis [39]. In our study, *MIR*502 was down-regulated in ovarian cancer, and the expression of CCND1 was negatively correlated with *MIR502*, which means CCND1 is overexpressed in OC. In addition, the PPI network showed that CCND1 plays a core function in interacting with other proteins, which further verified the important role of CCND1 in regulating the progression of OC. The development of OC may be slowed down by up-regulating* MIR502*, which decreases the expression of CCND1 and restrains the cell cycle.

The MYC family of proto-oncogenes is comprised of *c-*MYC, MYCN and MYCL [40]. c-MYC as an oncogene in numerous cancer cells plays an important role in a myriad of biological processes, including cell growth, cell cycle progression and proliferation [41, 42] by cooperating with YAP and activating a large number of target genes [43]. In fact, the amplification of *c*-MYC has been reported in ovarian cancer [44]. Previous studies showed that higher levels of *c*-MYC expression led to a faster recurrence and worse overall survival rate of patients with high grade serous ovarian cancer and was related to cisplatin resistance of ovarian cancer cells. Silencing of *c*-MYC inhibited the growth of cisplatin-resistant ovarian cancer. Thus, *c*-MYC targeted therapy is a potential treatment for ovarian cancer patients with high expression of *c*-MYC, including those who are resistant to cisplatin. This means that *c*-MYC may act as a new biomarker and therapy target for the chemotherapy response. Another member of the MYC family, MYCN, controls the basic process of embryonic development. MYCN signalling disorders leads to a variety of tumours, including neuroblastoma, medulloblastoma, rhabdomyosarcoma, Wilms tumour, prostate cancer and lung cancer. In neuroblastoma, a genetic aberration of MYCN amplification is related to a poor prognosis and failure of therapy. MYCN targeted therapy has been proposed as a new strategy for cancer treatment, and many effort has been made to develop direct and indirect MYCN inhibitors with potential clinical applications [45].

FGF1 belongs to the fibroblast growth factors (FGFs) family, whose function is regulating many cellular processes, including cell proliferation, differentiation and survival as an oncogene [46–48]. FGF1 is associated with tumour development, as it is upregulated in various cancers, including breast cancer, gliomas and ovarian cancer. The expression of FGF1 has a strong relationship with a poor prognosis and chemoresistance of tumours [49–52]. FGF1 has been considered as a potential prognostic marker for OC [53]. Compared with other family members, FGF1 genetic variation has the most significant correlation with an increased risk of ovarian cancer [54]. In addition, FGF1 expression is also an important determinant of survival and response to platinum chemotherapy. Therefore, the regulation of FGF1 by different mechanisms may play an important role in the development of ovarian cancer [55]. Our study suggested that *MIR502* had a counter-regulatory expression effect on FGF1, and a low level of *MIR502* expression increases FGF1 expression in ovarian cancer, which may lead to OC development and platinum chemotherapy resistance.

GLI family zinc finger proteins mediate Sonic hedgehog (Shh) signalling, and they exist in embryonic tumour cells as effective oncogenes. The proteins encoded by GLI2 belong to the C2H2-type zinc finger protein subclass of the GLI family. Researchers have found that the expression of GLI2 is regulated by Yap/TAZ, which activates the downstream regulatory factors of Shh signalling and promotes proliferation [56]. A large body of evidence has implicated GLI2 as a key regulator link in the cell cycle. Nagao et al. reported that silencing the expression of GLI2 made the cell cycle stop in G1 phase, which prevented the growth of osteosarcoma [57]. Similar mechanisms have been reported in human vascular smooth muscle cells [58] and myofibroblasts [59]. The same thing was observed in cervical cancer, that overexpression of GLI2 increased proliferation. All of the research has demonstrated that GLI2 promoted cell proliferation and exerted a tumour-promoting role in cancer. In our study, GLI2 as a downstream target of the Hippo signalling pathway was highly expressed due to the negative regulation by *MIR*502, resulting in an acceleration of the pathological process of ovarian cancer. GLI2 may be targeted as a novel therapeutic strategy in the future.

In summary, we have discovered that *MIR*502 expression in ovarian cancer is lower than that in normal tissue, which means that *MIR*502 acts as a significant tumour suppressor in ovarian cancer. *MIR*502 expression level was also correlated with ovarian cancer overall survival outcomes. Additionally, our analysis showed that the expression of *MIR*502 was regulated by NRF1 and further induced apoptosis and inhibiting proliferation by regulating genes downstream of the Hippo signalling pathway, including CCND1, FGF1, MYC and GLI2. In our study, we propose novel mechanisms between *MIR*502 and ovarian cancer that have not been elucidated previously. The immediate application of our findings is that *MIR*502 can be used as a prognostic tool in ovarian cancer. A better result is that our research on *MIR*502 in ovarian cancer will promote more extensive research on the molecular mechanisms of *MIR*502 and provide a reference for improving the clinical treatment of ovarian cancer.

## Conclusion

Our results suggested that *MIR*502 might be modulated by NRF1 and function as a potential tumour suppressor by regulating the Hippo signalling pathway, which regulates downstream anti-apoptosis and pro-proliferation genes, therefore providing a novel candidate for developing *MIR*502-based therapeutic strategies.

## Data Availability

The microRNA expression datasets used in this study (GEO: GSE83693 and GSE119055) were acquired from the National Center for Biotechnology Information (NCBI) Gene expression (http://www.ncbi.nlm.nih.gov/geo/).
